# Crosstalk between hypoxia-inducible factor (HIF) and lncRNAs in digestive tumors: from molecular mechanisms to clinical translation

**DOI:** 10.3389/fcell.2025.1611889

**Published:** 2025-08-08

**Authors:** Lifeng Gan, Peiyue Luo, Junrong Zou, Wei Li, Qi Chen, Le Cheng, Fangtao Zhang, Haidong Zhong, Yiran Lu, Liying Zheng, Biao Qian

**Affiliations:** ^1^ The First Clinical College, Gannan Medical University, Ganzhou, Jiangxi, China; ^2^ Department of Urology, The First Affiliated hospital of Gannan Medical University, Ganzhou, Jiangxi, China; ^3^ Department of Urology and Andrology, The Key Laboratory of the First Clinical Medical College of Gannan Medical University, Ganzhou, Jiangxi, China; ^4^ Department of Graduate, The First Affiliated Hospital of Gannan Medical University, Ganzhou, Jiangxi, China

**Keywords:** hypoxia, HIF, lncRNA, digestive system tumors, liver cancer, colorectal cancer, gastric cancer, pancreatic cancer

## Abstract

Hypoxia is a characteristic feature of the tumor microenvironment that significantly influences cancer progression and treatment responses. Hypoxia-inducible factor (HIF), a key regulator of hypoxic adaptation, has been demonstrated to modulate hypoxic gene expression profiles and signaling networks, thereby serving as a potential therapeutic target. Long-stranded non-coding RNAs (lncRNAs), defined as non-coding RNAs exceeding 200 nucleotides in length, regulate various cellular processes by modulating gene expression at transcriptional, post-transcriptional, and epigenetic levels. Evidence suggests that lncRNAs can be regulated by HIF at the transcriptional level. Conversely, HIF itself can be modulated by numerous lncRNAs, with alterations in these lncRNAs being associated with tumorigenesis, resulting in a reciprocal regulatory network. Recently, the critical role of lncRNAs in hypoxia-driven cancer progression has been elucidated in digestive tumors, including colorectal, pancreatic, gastric, and hepatocellular carcinomas. An increasing number of studies have revealed the complex interplay between lncRNAs and HIF in regulating various processes such as proliferation, metastasis, apoptosis, and drug resistance. In this paper, we aim to provide a comprehensive summary of recent advances regarding the roles of hypoxia and lncRNAs in digestive system tumors and to illustrate the mechanisms through which lncRNAs interact with hypoxia in tumor cells. This will enhance our understanding of the regulatory roles of lncRNAs in modulating the microenvironment of digestive system tumors, thereby facilitating the development of novel anticancer drugs.

## Introduction

Tumors of the digestive system, particularly colorectal cancer (CRC), gastric cancer (GC), and liver cancer (LC), account for over 4 million new cases worldwide. These cancers rank among the ten leading causes of cancer-related deaths (Bray et al., 2024; Chandarana et al., 2024). The choice of treatment typically depends on the stage of the disease and may include surgery, chemotherapy, radiotherapy, and targeted therapy (Wang et al., 2018; Lee et al., 2018). However, the effectiveness of these treatments is often limited. Consequently, there is an urgent need to identify new therapeutic targets to improve the clinical management of digestive tumors (Wang et al., 2018).

Long non-coding RNAs are currently defined as a large, heterogeneous class of regulatory transcripts that exceed 200 nucleotides in length and lack significant protein-coding potential (Rinn and Chang, 2012). This extensive class encompasses various categories of transcriptional elements, including long intergenic and intronic ncRNAs, transcribed superconserved regions (TCRs), pseudogenes, enhancer RNAs (eRNAs), and antisense RNAs (asRNAs) (Kung et al., 2013). Although less than 1% of these RNAs are functionally annotated, mounting evidence indicates that lncRNAs play crucial roles in multiple stages of gene expression regulation, such as imprinting, transcription, RNA interference, RNA splicing, and translational control (Moran et al., 2012; Lee, 2012; Yang et al., 2014). In recent years, numerous dysregulated lncRNAs have been linked to various diseases, including cancer (Spizzo et al., 2012; Hu et al., 2023; Dashtaki and Ghasemi, 2023). The mechanisms by which lncRNAs function in cancer primarily involve regulating gene expression and cellular processes through multiple molecular pathways, thereby promoting tumorigenesis, progression, and drug resistance. These mechanisms can be summarized as follows: Competitive endogenous RNA (ceRNA) mechanism: lncRNAs can bind to miRNAs, acting as competitive endogenous RNAs, thereby regulating the expression of downstream target genes and influencing cancer cell proliferation and metastasis (Sun et al., 2016). This mechanism drives tumor progression by relieving miRNA inhibition of oncogenes or tumor suppressor genes; Epigenetic regulation mechanism: lncRNAs mediate histone modifications (such as methylation or acetylation) to regulate the transcriptional activity of key genes, such as those involved in epithelial-mesenchymal transition (EMT) regulation, thereby promoting cancer cell invasion and metastasis (Terashima et al., 2018); Signal pathway regulation mechanism: lncRNAs can interfere with multiple cellular signaling pathways (such as Wnt or VEGF signaling) by affecting the activity of protein kinases or transcription factors, thereby regulating cancer cell metabolism, survival, and migration (Li et al., 2022); Drug resistance regulation mechanism: lncRNAs regulate the expression of genes related to drug targets or apoptosis pathways, leading to chemotherapy resistance and playing a key role in cancer progression (Wang J. et al., 2021). Overall, lncRNAs act as indispensable regulatory factors in cancer pathogenesis through comprehensive mechanisms such as ceRNA, epigenetic regulation, and signaling pathways. These mechanisms reveal the potential value of lncRNAs in diagnosis and treatment.While some cancer-related lncRNAs have been well-characterized (Huarte, 2015), the functions of the majority remain largely unknown. The dysregulation of many cancer-associated lncRNAs correlates with clinicopathologic features and survival outcomes of patients, suggesting that functional annotation of these lncRNAs could ultimately lead to new avenues for early diagnosis and treatment of cancer (Qiu et al., 2013). Studies indicate that lncRNA regulation in response to hypoxia may play a pivotal role in the hypoxia-inducible factor (HIF) signaling cascade (Chang et al., 2016).

**FIGURE 1:**
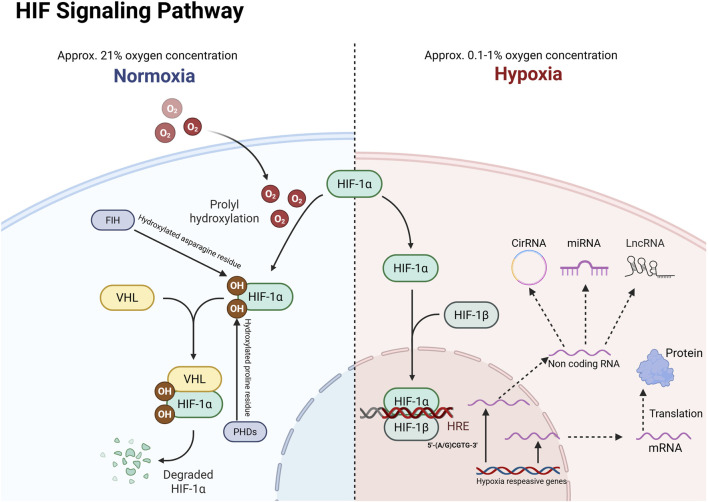
HIF-1α responds to gene transcription under hypoxic activation. VHL: Von Hippel-Lindau; HRE: hypoxia response element; PHD: Prolyl hydroxylase; FIH: Factor-inhibiting HIF.

HIF is a heterodimer composed of basic helix-loop-helix and PERN-ARNT-SIM (bHLH-PAS) family proteins, consisting of an inducible oxygen-regulated α-subunit and a stabilizing constitutive β-subunit (Edwards and Gorelick, 2022). In mammals, the α-subunit is encoded by three genes: HIF1A, HIF2A (also known as EPAS1, endothelial PAS structural domain-containing protein 1), and HIF3A. The HIF1β-subunit (HIF1B; also known as ARNT, the aryl hydrocarbon receptor nucleotide transporter) is encoded by two genes, ARNT1 and ARNT2. HIF-1α and HIF-2α regulate independent but overlapping sets of transcriptional target genes, while some splice variants of HIF-3α exert a dominant negative effect on HIF-dependent gene transcription (Zhuang et al., 2024). Under normoxic conditions, both HIF-1α and HIF-2α interact with FIH through their C-terminal transactivation domains (CAD), where FIH hydroxylates specific asparagine (Asn) residues [Asn803 in human HIF-1α (hsHIF-1α)], thereby inhibiting transcriptional activity by preventing the recruitment of p300/CBP coactivators. Additionally, PHD-mediated prolyl hydroxylation of HIF-α proteins promotes Von Hippel Lindau protein (VHL)-mediated ubiquitination and rapid proteasomal degradation. The combined activity of FIH and PHD ensures strict inhibition of HIF transcriptional activity under normoxic conditions.When hypoxia occurs (Usually refers to an oxygen concentration of approximately 0.1%–1%), HIF-1α subunits accumulate rapidly due to the inhibition of PHD dioxygenase activity, after which they translocate from the cytoplasm to the nucleus. In the nucleus, they interact with HIF-1β, CBP (CREB-binding protein), and p300 to form the HIF-1 transcriptional complex, which ultimately binds to the promoter region of HIF-1α target genes. This binding triggers a series of cellular hypoxic adaptations, including enhanced cell proliferation, angiogenesis, decreased apoptosis, increased autophagy, and enhanced invasion and metastasis (Semenza, 2014; Semenza, 2010). In addition to the protein-coding transcriptome, an increasing body of research indicates that the non-coding transcriptome also responds to hypoxia, playing various critical roles in cancer progression and metastasis in this context (Choudhry et al., 2016; Daskalaki et al., 2018; Yin and Ma, 2024) (Figure 1).

The mechanisms by which lncRNAs interact with hypoxia-inducible factors (HIFs), particularly HIF-1α and HIF-2α, encompass direct regulation, stability modulation, transcriptional co-activation, and feedback loops. For instance, LncHIFCAR (formerly known as MIR31HG) activates hypoxia-related signaling pathways, such as glycolysis and metabolic reprogramming, and promotes tumor growth and metastasis by directly binding to HIF-1α to form a complex. This interaction facilitates the recruitment of HIF-1α with the coactivator p300, thereby enhancing the binding affinity of HIF-1α to the promoters of target genes (Shih et al., 2017); Additionally, LncRNA RP11-390F4.3 enhances the interaction between HIF-1α and ZMIZ1 (a HIF-1α coactivator) through METTL4-mediated RNA methylation (6 mA modification), which activates EMT-related genes and drives tumor metastasis (Hsu et al., 2022); Furthermore, LncRNA HISLA, secreted by tumor-associated macrophages, promotes tumor growth and metastasis by inhibiting the interaction between prolyl hydroxylase PHD2 and HIF-1α, thus preventing hydroxylation and VHL-mediated ubiquitin degradation of HIF-1α. This stabilization of HIF-1α enhances glycolysis and apoptosis resistance in tumor cells (Chen et al., 2019); Under hypoxic conditions, HIF-1α directly binds to the promoters of certain lncRNAs (e.g., H19, DARS-AS1) and induces their transcription. For example, HIF-1α binds to the H19 promoter to promote its expression, while H19 further stabilizes the HIF-1α protein, creating a positive feedback loop (Xie et al., 2023); Finally, the long non-coding RNA (LncRNA) LINK-A further influences the regulation of the FTO/LINK-A/MCM3/HIF-1α signaling pathway by disrupting the binding of MCM3 (an HIF-1α inhibitor) to HIF-1α, thereby activating glycolysis and chemotherapy resistance, and alleviating the transcriptional suppression exerted by MCM3 (Nan et al., 2023). In summary, the intricate crosstalk between HIF and lncRNAs represents a pivotal regulatory axis in digestive tumor pathogenesis, warranting comprehensive exploration of its molecular underpinnings and clinical implications.

## Interaction of lncRNA and HIF signaling in digestive system tumors

Based on current research progress, lncRNAs derived from exosomes play a systemic regulatory role in the hypoxic microenvironment of gastrointestinal tumors, promoting tumor proliferation, metastasis, and immune evasion through intercellular transfer (Xue et al., 2017). In the identification of novel hypoxia-responsive lncRNAs, novel lncRNAs such as RP11-390F4.3, which are HIF-targeted transcripts, have been shown to be directly activated by HIF-1α, driving tumor invasion by inducing EMT (Peng et al., 2020). Notably, the lncRNA-HIF interaction network simultaneously coordinates metabolic reprogramming and immune suppression in the microenvironment, including: (1) HALs (hypoxia-associated lncRNAs) enhance glycolysis and activate autophagy through HIF signaling (Huang et al., 2019); (2) the ncRNA/HIF-1α interaction network regulates the expression of immune checkpoint molecules such as PD-L1, promoting T cell functional exhaustion (Cowman and Koh, 2022; You et al., 2021). Regarding clinical translation strategies, extracellular lncRNAs and cellular HALs can serve as diagnostic markers or therapeutic targets. Targeted interventions, such as RNA nanomedicines, can block the HIF signaling pathway, or liquid biopsy can be used to monitor microenvironment hypoxia status (Huang et al., 2019).

In recent years, studies examining the interaction between HIFs and lncRNAs in digestive system tumors have garnered increasing attention. For instance, one study highlighted that the positive feedback loop involving HIF-1α, lncRNA ZEB1-AS1, ZEB1, and HDAC1 contributes to the hypoxia-induced oncogenicity and metastasis of pancreatic cancer (PC), indicating its potential as a novel therapeutic target for PC (Jin et al., 2021).Additionally, the combination of elevated lncRNA TRERNA1 and HIF-1α, alongside reduced E-calmodulin, predicts poor prognosis in patients with hepatocellular carcinoma (HCC) (Qian et al., 2021).Furthermore, LINC00152, which may function as a competing endogenous RNA, enhances the translation of HIF-1 in the cytoplasm of hypoxic colorectal cancer cells (Nishizawa et al., 2018). This paper aims to summarize the current understanding of the regulatory roles of hypoxia-responsive lncRNAs, emphasizing their inter-regulation with HIFs, modulation of the hypoxia response, and correlation with clinical features of digestive tumors. These insights may position lncRNAs as molecular markers for disease diagnosis, prognosis, and evaluation of therapeutic efficacy.

## Interaction of lncRNA and HIF signaling in hepatocellular carcinoma

Recent studies have underscored the significant role of hypoxia-associated lncRNAs in HCC (Table 1). These lncRNAs participate in various processes, including glucose metabolism, cancer stem cell maintenance, apoptosis, proliferation, and immune evasion, all of which contribute to the poor prognosis of HCC patients. Specifically, lncRNA MRVI1-AS1 enhances SKA1 expression in hepatocellular carcinoma by binding to the RNA-binding protein CELF2, thereby facilitating its interaction with the downstream target gene SKA1 mRNA, which stabilizes SKA1 and activates its expression. Hypoxia induces MRVI1-AS1 expression in a HIF-1-dependent manner, and MRVI1-AS1 stabilizes SKA1 mRNA through a CELF2-dependent mechanism, promoting liver cancer cell proliferation and invasion (Tuo et al., 2023) (Table 1). Furthermore, one study identified HLA complex group 15 (HCG15) as a novel hypoxia-associated lncRNA in HCC cells, with both hypoxia and HIF prolyl hydroxylase inhibitors significantly increasing HCG15 expression in these cells. HCG15 enhances the migration and invasion of HCC cells by promoting the transcription of zinc finger protein 641 (ZNF641) through the upstream transcription factor 1 (USF1), thereby facilitating HCC cell migration, invasion, and proliferation (Yan et al., 2022). In a hypoxic environment, HIF-1α promotes its transcription by binding to the promoter of lncRNA HMMR-AS1, which induces an increase in the number of secreted exosomes. HMMR-AS1 can competitively bind with miR-147a, thereby preventing the degradation of ARID3A and promoting macrophage M2 polarization, which further accelerates tumor growth in HCC (Wang X. et al., 2022). HIF-1α activates the transcription of KDM4A-AS1 by directly binding to its promoter region, resulting in high expression levels of KDM4A-AS1 in HCC. The elevated KDM4A-AS1 expression competitively binds to miR-411-5p, relieving its inhibitory effect on the nuclear transporter protein KPNA2. This activation of the KPNA2/AKT signaling pathway leads to continuous phosphorylation of AKT (Chen et al., 2021), which not only enhances the proliferation and invasive capabilities of liver cancer cells but also promotes their metastatic potential by inducing the upregulation of epithelial-mesenchymal transition-related markers, such as N-cadherin and Vimentin (Cao et al., 2024). Additionally, lncRNA NEAT1 forms a positive feedback loop with HIF-1α, up-regulating NEAT1 expression in the hypoxic microenvironment. NEAT1 further enhances hypoxia signaling by stabilizing HIF-1α mRNA, collectively driving malignant phenotypes of HCC cells, including proliferation, invasion, and resistance to apoptosis (Su et al., 2023; Liu T. et al., 2022; Yang et al., 2023). Zhang et al. demonstrated that under hypoxic conditions, HIF-1α activates the expression of lncRNA NEAT1 through transcriptional regulation, while NEAT1 promotes the transcription of PKM2 by binding to FOXP3 protein, thereby enhancing the glycolytic metabolism and proliferation of HCC cells (Zhang X. et al., 2020).

**TABLE 1:** Interaction of lncRNA and HIF signaling in hepatocellular carcinoma.

HIF→LncRNA OR LncRNA→HIF		Impact on target	Functional impact	Pro/Anti tumor	Refs
HIF-1α	MRVI1-AS1	Upregulated	Promote metastasis and growth	Pro-tumor	Tuo et al. (2023)
HIF-1α	HCG15	Upregulated	Promote proliferation and migration	Pro-tumor	Yan et al. (2022)
HIF-1α	HMMR-AS1	Upregulated	Promote tumor growth	Pro-tumor	Wang et al. (2022a)
HIF-1α	KDM4A-AS1	Upregulated	Promote metastasis and growth	Pro-tumor	Chen et al. (2021)
HIF-1α	NEAT1	Upregulated	Promotes cell viability, migration and invasion and inhibits apoptosis	Pro-tumor	Zhang et al. (2020a)
HIF-1α	DACT3-AS1	Upregulated	Promote cell transfer	Pro-tumor	Wang et al. (2022b)
HIF-1α	NPSR1-AS1	Upregulated	Promotes proliferation and glycolysis	Pro-tumor	He et al. (2020)
HIF-1α	NEAT1	Upregulated	Maintains cell growth and inhibits apoptosis and cell cycle arrest	Pro-tumor	Zhang et al. (2020b)
HIF-1α	RAET1K	Upregulated	Promote proliferation and invasion	Pro-tumor	Zhou et al. (2020)
MAPKAPK5-AS1	PLAGL2/HIF-1α	Upregulated	Promotes HCC cell growth *in vivo* and lung metastasis	Pro-tumor	Wang et al. (2021b)
FRMD6-AS1	SENP1/HIF-1α	Upregulated	Promotes migration and stemness	Pro-tumor	Sun et al. (2023)
ZFPM2-AS1	miR-576-3p/HIF-1α	Upregulated	Promote proliferation, migration and invasion	Pro-tumor	Song et al. (2021)
NEAT1	HIF-2α	Upregulated	Promotes tumor cell EMT, migration, and invasive capacity	Pro-tumor	Zheng et al. (2018)
CPS1-IT1	HIF-1α	Downregulated	Inhibition of EMT progression and metastasis, proliferation, migration and invasive ability	Anti-tumor	Wang et al. (2017), Wang et al. (2016)
MIR155HG	HIF-1α	Upregulated	Promoting immune escape	Pro-tumor	Qiu et al. (2024)
PAARH	HIF-1α/VEGF	Upregulated	Promotes HCC progression and angiogenesis	Pro-tumor	Wei et al. (2022)
MIAT	HIF-1α	Upregulated	Promote proliferation, migration and invasion	Pro-tumor	Liu et al. (2020)
HOTAIR	HIF-1α	Upregulated	Promote glycolysis	Pro-tumor	Hu et al. (2020)
UBE2CP3	ERK/HIF-1α/p70S6K/VEGFA	Upregulated	Promotes proliferation, migration and angiogenesis	Pro-tumor	Lin et al. (2018)
p21	HIF-1α	Downregulated	Inhibits proliferation and migration, promotes apoptosis	Anti-tumor	Ye et al. (2019)
SZT2-AS1	HIF-1α/HIF-1β	Forms HIF-1 heterodimers	Promotes HCC growth, metastasis and angiogenesis	Pro-tumor	Liu et al. (2024a)

According to current research, the promoter regions of lncRNAs such as HIF1A-AS3, SZT2-AS1, DARS-AS1, and HOTAIR have been identified as containing hypoxia response elements. As a transcription factor, HIF-1α/HIF-1β binds to the promoters of lncRNAs and regulates various cellular processes. Additionally, tumor-associated lncRNAs can modulate the activity of HIF-1α, ultimately exerting either pro- or anti-tumorigenic effects on tumor cells. Studies indicate that HIF-1α in the hypoxic microenvironment may positively feedback to upregulate its own expression by directly binding to the promoter region of lncRNA MAPKAPK5-AS1. This interaction enhances the transcriptional activity of HIF-1α by stabilizing the PLAGL2 protein, thereby forming a MAPKAPK5-AS1/PLAGL2/HIF-1α signaling loop that promotes the malignant phenotype of hepatocellular carcinoma cells, including proliferation, EMT, and metastasis (Wang L. et al., 2021). lncRNA FRMD6-AS1 is significantly upregulated in hepatocellular carcinoma tissues and cells, regulating the stability of HIF-1α through direct interaction with the de-SUMOylating enzyme SENP1. The binding of FRMD6-AS1 to SENP1 was confirmed by RNA immunoprecipitation, which promotes HIF-1α de-SUMOylation, thereby enhancing its protein stability and activating downstream hypoxia signaling pathways. Consequently, HIF-1α, as a core transcription factor, is activated by the FRMD6-AS1/SENP1 axis, further driving the proliferation and invasion of HCC (Sun et al., 2023). Compared to normal liver tissues, the expression of lncRNA ZFPM2-AS1 is significantly upregulated in HCC tissues. ZFPM2-AS1 can bind to miR-576-3p, positively regulating the expression of the miR-576-3p target gene HIF-1α through miR-576-3p adsorption, thereby promoting the proliferation, migration, and invasion of HCC cells (Song et al., 2021). Additionally, the expression levels of lncRNAs NEAT1 and HIF-2α are significantly increased in HCC tissues and cell lines. *In vitro* assays demonstrated that NEAT1 enhances tumor cell EMT, migration, and invasion by stimulating HIF-2α activation in HCC (Zheng et al., 2018). Wang et al. reported a reduction in the expression of lncRNA CPS1-IT1 in hepatocellular carcinoma, noting its ability to interact with heat shock protein 90 (Hsp90), a critical protein in HCC. Their findings indicate that CPS1-IT1 reduces the binding affinity between Hsp90 and HIF-1α, leading to decreased activation of HIF-1α and significantly inhibiting HCC cell proliferation, migration, and invasiveness. The inactivation of HIF-1α results in the downregulation of EMT-related proteins, thereby inhibiting EMT (Wang et al., 2016). In another experiment, it was found that the promoter region of CPS1-IT1 contains a potentially evolutionarily conserved binding site for FOXA2. Furthermore, melatonin was shown to induce the expression of lncRNA CPS1-IT1 by upregulating FOXA2 expression, which subsequently inhibited HCC progression through CPS1-IT1-mediated inactivation of HIF-1α (Wang et al., 2017). Collectively, these findings establish hypoxia-responsive lncRNAs as critical modulators of HCC progression through HIF-dependent regulation of metastasis, metabolism, and immune evasion, highlighting their potential as therapeutic targets.

## Interaction of lncRNA and HIF signaling in colorectal cancer

Colorectal cancer ranks as the third most prevalent cancer and the second leading cause of cancer-related mortality globally, with 1,142,286 new cases documented in 2022 (Baidoun et al., 2021). Recent research has focused on developing effective diagnostic and therapeutic strategies for CRC (Table 2). Thus, it is imperative to thoroughly comprehend the molecular mechanisms that govern the initiation and progression of CRC. Within the context of colorectal cancer, the hypoxic microenvironment triggers the transcriptional upregulation of lncRNA STEAP3-AS1 by HIF-1α, which stabilizes the STEAP3 protein and activates the Wnt/β-catenin signaling pathway through direct interaction with its adjacent protein-coding gene, STEAP3. The interaction between STEAP3-AS1 and STEAP3 enhances the nuclear translocation of β-catenin, promotes the transcription of downstream oncogenes (such as c-Myc and Cyclin D1), and ultimately facilitates the proliferation and metastasis of CRC cells (Zhou et al., 2022) (Table 2). Furthermore, lncRNA MNX1-AS1 is highly expressed in colorectal adenocarcinoma (COAD) tissues and forms a functional complex with PPFIA4, leading to the phosphorylation of AKT and the activation of the HIF-1 signaling pathway. This activation drives the stemness characteristics of COAD cells, as evidenced by their enhanced self-renewal capacity and drug resistance. Consequently, MNX1-AS1 promotes the stemness, proliferation, and migration of colorectal cancer cells while inhibiting apoptosis through the activation of the PPFIA4-mediated AKT/HIF-1 signaling pathway, thereby contributing to tumor progression (Sun et al., 2022). One study demonstrated that LncRNA LINC00525 is up-regulated in CRC and promotes UBE2Q1 expression by binding to miR-338-3p. This interaction leads to the stabilization of β-catenin, which enhances hypoxia-induced glycolysis through the activation of HIF-1α (Meng et al., 2022). Additionally, Yu et al. reported that lncRNA COL4A2-AS1 is also upregulated in CRC tissues and cells. Silencing of lncRNA COL4A2-AS1 significantly inhibited CRC cell viability, proliferation, and aerobic glycolysis, while inducing apoptosis and reducing tumor volume and weight. Furthermore, COL4A2-AS1 upregulated HIF1A by down-regulating miR-20b-5p, thereby promoting CRC cell proliferation (Yu et al., 2021). Conversely, the expression of lncRNA CPS1-IT1 was significantly decreased in CRC tissues and cell lines, while the levels of HIF-1α and LC3-II were increased. Overexpression of CPS1-IT1 inhibited the activation of HIF-1α, which subsequently suppressed hypoxia-induced autophagy, thereby inhibiting CRC cell metastasis and EMT (Zhang et al., 2018). Taken together, lncRNA-HIF networks drive CRC malignancy by orchestrating stemness, glycolytic reprogramming, and autophagy pathways, positioning them as promising biomarkers for clinical intervention.

**TABLE 2:** Interaction of lncRNA and HIF signaling in colorectal cancer.

HIF→LncRNA OR LncRNA→HIF		Impact on target	Functional impact	Pro/Anti tumor	Refs
HIF-1α	STEAP3-AS1	Upregulated	Promoting proliferation and metastasis	Pro-tumor	Zhou et al. (2022)
HIF-1α	LINC00511	Upregulated	Promotes proliferation and reduces apoptosis	Pro-tumor	Sun et al. (2020a)
MNX1-AS1	AKT/HIF-1α	Upregulated	Promotes stemness, proliferation and migration and inhibits apoptosis	Pro-tumor	Sun et al. (2022)
LINC00525	HIF-1α	Upregulated	promote proliferation	Pro-tumor	Meng et al. (2022)
COL4A2-AS1	miR-20b-5p/HIF-1α	Upregulated	Promotes proliferation and glycolysis	Pro-tumor	Yu et al. (2021)
CPS1-IT1	HIF-1α	Downregulated	Inhibition of autophagy, metastasis and EMT	Anti-tumor	Zhang et al. (2018)
CRNDE	HIF-1α	Upregulated	Promote tumor growth and metastasis	Pro-tumor	Tang et al. (2022)
XIST	HIF-1α	Upregulated	Promotes EMT, migration and proliferation	Pro-tumor	Yang et al. (2020)

## Interaction of lncRNA and HIF signaling in gastric cancer

Gastric cancer is the most prevalent tumor of the gastrointestinal tract and ranks among the five leading causes of cancer-related mortality (Table 3). According to GLOBOCAN 2022, there were 968,784 reported cases globally (Bray et al., 2024). Liu et al. identified aberrant expression of lncRNA BC005927 in hypoxia-induced gastric cancer cells and tissues. They further demonstrated that the interaction between HIF-1α and HIF-1α-responsive elements in the promoter region of BC005927 induces its expression. EPHB4 is located 300 kb upstream of BC005927, and its expression is positively correlated with that of BC005927 in gastric cancer tissues. The up-regulation of BC005927 enhances EPHB4 expression, which in turn promotes gastric cancer metastasis under hypoxic conditions (Liu et al., 2018) (Table 3). Additionally, both HIF-1α and lncRNA GAPLINC are upregulated in gastric cancer tissues and cell lines. HIF-1α binds to the hypoxia response element (HRE) of the GAPLINC promoter, thereby increasing promoter activity in human MKN45 and SGC7901 cells. The overexpression of GAPLINC in gastric cancer tissues promotes hypoxia-induced tumor proliferation, migration, and invasive behavior *in vivo* (Liu et al., 2016). The high expression of NUTM2A-AS1 has been detected in HGC-27 and SNU-1 cells. miR-376a is a newly discovered microRNA that interacts with NUTM2A-AS1, which inhibits the expression of miR-376a. miR-376a binds to the 3′-UTR region of TET1 and HIF-1A, thereby inhibiting their expression levels. The interaction between TET1 and HIF-1A positively regulates PD-L1, and the overexpression of PD-L1 enhances the function of the NUTM2A-AS1/miR-376a axis in treating malignant tumors in gastric cancer (Wang et al., 2020). ZEB2-AS1 is a conserved natural antisense transcript corresponding to the 5′-UTR of ZEB2, which has been implicated in the EMT process, closely related to carcinogenesis (Gao et al., 2018). ZEB2-AS1 functions as a ceRNA in the regulation of gastric cancer progression. miR-143-5p is a potential target of ZEB2-AS1. Bioinformatics analyses have predicted that HIF-1α is a target of miR-143-5p. In the study by Wu et al., the expression of HIF-1α was negatively regulated by miR-143-5p and was positively correlated with the expression level of ZEB2-AS1. ZEB2-AS1 was found to be upregulated in gastric tissues and cell lines, promoting cell proliferation and metastasis through the miR-143-5p/HIF-1α pathway (Wu et al., 2019). Overall, HIF-induced lncRNAs promote GC aggressiveness via EMT activation and chemoresistance pathways, underscoring their role as key mediators of hypoxia-driven tumor evolution.

**TABLE 3:** Interaction of lncRNA and HIF signaling in gastric cancer.

HIF→LncRNA OR LncRNA→HIF		Impact on target	Functional impact	Pro/Anti tumor	Refs
HIF-1α	BC005927	Upregulated	Promote transfer	Pro-tumor	Liu et al. (2018)
HIF-1α	GAPLINC	Upregulated	Promoting migration and invasion	Pro-tumor	Liu et al. (2016)
HIF-1α	HYPAL	Upregulated	Promotes proliferation and inhibits apoptosis	Pro-tumor	Piao et al. (2022)
HIF-1α	PCGEM1	Upregulated	Promote invasion and metastasis	Pro-tumor	Zhang et al. (2019)
NUTM2A-AS1	HIF-1α	Upregulated	Promoting gastric cancer cell viability, invasion and drug resistance	Pro-tumor	Wang et al. (2020)
ZEB2-AS1	HIF-1α	Upregulated	Promote proliferation, growth and invasion	Pro-tumor	Wu et al. (2019)

## Interaction of lncRNA and HIF signaling in pancreatic cancer

Pancreatic cancer is the fourth leading cause of cancer-related deaths, with both its incidence and mortality rates rising in recent years (Table 4). A significant challenge in managing this disease is that most patients remain asymptomatic during its progression. Consequently, effective diagnosis relies on the identification and screening of high-risk individuals prior to the onset of symptoms (Kolbeinsson et al., 2023). In pancreatic cancer, the hypoxic microenvironment activates HIF-1α, which binds directly to the HRE1-binding site in the promoter region of lncRNA LINC00460, thereby promoting its transcription. LINC00460 functions as a sponge for miR-4689, leading to the upregulation of UBE2V1, a protein that regulates both the proliferation and metastasis of pancreatic cancer, as well as the stability of p53. Additionally, LINC00460 enhances tumorigenesis by sequestering USP10, which alters the stability and subcellular localization of p53, further promoting pancreatic cancer proliferation and metastasis (Zhang R. et al., 2023) (Table 4). The expression of lncRNA NR2F1-AS1 is elevated in pancreatic cancer tissues and cell lines, and NR2F1 is both a target and positively regulated by NR2F1-AS1. Hypoxia significantly enhances the transcription of NR2F1-AS1-WT, an effect that is inhibited by HIF-1α knockdown. Moreover, NR2F1-AS1 promotes the proliferation and invasion of pancreatic cancer cells through NR2F1-mediated activation of the AKT/mTOR signaling pathway (Liu Y. et al., 2022). Jiang et al. demonstrated that the transcription of lncRNA-BX111 is induced by HIF-1α in response to hypoxia. BX111 activates the transcription of ZEB1, a critical regulator of epithelial-mesenchymal transition, by recruiting the transcription factor Y-box binding protein (YB1) to its promoter region. Thus, HIF-1α-induced BX111 promotes the proliferation and invasion of pancreatic cancer cells via the activation of ZEB1 transcription (Deng et al., 2018). LncRNA ENST00000480739 is downregulated in pancreatic ductal adenocarcinoma (PDAC). This lncRNA is located on chromosome 12, upstream of the OS-9 promoter region, and its low expression is closely associated with TNM staging and metastasis. ENST00000480739 suppresses pancreatic cancer by inhibiting HIF-1α expression through the regulation of OS-9 function in the invasion of pancreatic cancer (Sun et al., 2014). Conversely, lncRNA PCED1B-AS1 is upregulated in pancreatic cancer tissues and cells. It acts as a ceRNA in PDAC progression by negatively regulating miR-411-3p expression, which targets HIF-1α. PCED1B-AS1 regulates the miR-411-3p/HIF-1α axis to reduce PDAC cell proliferation, invasion, and EMT (Zhang et al., 2021). Furthermore, overexpression of lncRNA MTA2TR is associated with the progression of pancreatic cancer. MTA2TR activates MTA2 transcription by recruiting ATF3 to the MTA2 promoter, while hypoxia-induced MTA2TR stabilizes the HIF-1α protein by promoting its deacetylation, further enhancing HIF-1α transcriptional activity. The feedback loop between MTA2TR and HIF-1α regulates the proliferation and invasion of pancreatic cancer cells (Zeng et al., 2019). Under hypoxic conditions, lncRNA BANCR expression is significantly upregulated in pancreatic cancer cells. BANCR promotes the expression of vascular endothelial growth factor C (VEGF-C) and its receptor, vascular endothelial growth factor receptor 3 (VEGFR-3), by stabilizing the transcriptional activity of HIF-1α. This mechanism stimulates the generation of micro-lymphatics in pancreatic cancer and enhances the invasive ability of tumor cells to the lymphatic system, ultimately leading to lymph node metastasis. This process is particularly significant in the hypoxic microenvironment, where HIF-1α, as a core transcription factor, directly mediates the regulation of lymphatic metastasis-related target genes by BANCR (Hao et al., 2024). In essence, PC-specific lncRNAs amplify HIF signaling to facilitate lymphangiogenesis, stemness, and metabolic adaptation, revealing novel avenues for overcoming therapeutic resistance.

**TABLE 4:** Interaction of lncRNA and HIF signaling in pancreatic cancer.

HIF→LncRNA OR LncRNA→HIF		Impact on target	Functional impact	Pro/Anti tumor	Refs
HIF-1α	linc00460	Upregulated	Promoting proliferation and metastasis	Pro-tumor	Zhang et al. (2023a)
HIF-1α	NR2F1-AS1	Upregulated	Promote proliferation, migration and invasion	Pro-tumor	Liu et al. (2022b)
HIF-1α	BX111	Upregulated	Promotes lymphatic infiltration and distant metastasis	Pro-tumor	Deng et al. (2018)
HIF-1α	NNT-AS1	Upregulated	Promoting immune escape	Pro-tumor	Lu et al. (2023)
HIF-1α	CF129	Downregulated	Promote invasion and metastasis	Pro-tumor	Liu et al. (2019)
HIF-1α	NUTF2P3-001	Upregulated	Promote proliferation and invasion	Pro-tumor	Li et al. (2016)
ENST00000480739	HIF-1α	Downregulated	Inhibition of tumor invasion	Anti-tumor	Sun et al. (2014)
PCED1B-AS1	HIF-1α	Upregulated	Promotes proliferation, invasion and EMT	Pro-tumor	Zhang et al. (2021)
MTA2TR	HIF-1α	Upregulated	Promote proliferation and invasion	Pro-tumor	Zeng et al. (2019)
BANCR	HIF-1α	Upregulated	Promote lymphangiogenesis, lymphatic transfer	Pro-tumor	Hao et al. (2024)
PVT1	HIF-1α	Upregulated	Promote proliferation and invasion	Pro-tumor	Sun et al. (2020b)

## Effect of interaction between lncRNA and HIF on digestive system tumors

The cellular processes regulated by the interaction between lncRNAs and HIF are diverse. However, most researchers have concentrated on proliferation, migration, invasion, EMT, and glycolysis, which are among the most extensively studied hallmarks of cancer (Hanahan, 2022). In contrast, other processes such as apoptosis, immune evasion, and angiogenesis have received comparatively less attention. In the following sections, we will elucidate the role of the interaction between lncRNAs and HIF across all these processes.

## Interaction between lncRNA and HIF regulates proliferation and apoptosis in digestive tumors

Cell cycle dysregulation is a fundamental mechanism underlying uncontrolled cell proliferation, which is a hallmark of cancer cells (Williams and Stoeber, 2012). Research has demonstrated that lincRNA-p21 is overexpressed in the highly metastatic hepatocellular carcinoma cell line MHCC97H. The findings indicate that the expression levels of HIF-1α protein and vascular endothelial growth factor (VEGF) are significantly downregulated, suggesting that HIF-1α is a target of lincRNA-p21, and that its downregulation results in decreased VEGF levels. Furthermore, overexpression of lincRNA-p21 notably inhibited the proliferation of MHCC97H cells and enhanced apoptosis levels (Ye et al., 2019). In another study focused on hepatocellular carcinoma, lncRNA-NEAT1 was identified as a hypoxia-responsive lncRNA in an *in vitro* HCC cell line. It was observed that under hypoxic conditions, the transcriptional upregulation of lncRNA-NEAT1 in HCC cells is mediated by HIF-1α. Under normoxic conditions, overexpression of lncRNA-NEAT1 did not affect the viability of SNU-182 cells; however, knockdown of lncRNA-NEAT1 resulted in inhibited cell viability. Additionally, cells transfected with pcDNA3.1-NEAT1 exhibited a higher proliferation rate under hypoxic conditions, while knockdown of lncRNA-NEAT1 suppressed proliferation. Consistently, knockdown of lncRNA-NEAT1 induced apoptosis in SNU-182 cells, whereas treatment with siRNA-NEAT1 increased the percentage of apoptotic cells, and overexpression of lncRNA-NEAT1 decreased the percentage of apoptotic cells after 24 h of exposure to hypoxic conditions (Zhang Q. et al., 2020). One study demonstrated that the transcription factor HIF-1α enhances the transcription of LINC00511 in colorectal cancer cells. The CCK-8 assay indicated that the knockdown of LINC00511 inhibited cellular proliferation, while its overexpression promoted proliferation. Furthermore, apoptosis analysis via flow cytometry revealed that LINC00511 knockdown increased apoptosis in CRC cells, whereas its overexpression decreased apoptosis. Additionally, chromatin immunoprecipitation (ChIP) and luciferase reporter gene assays indicated that HIF-1α promotes apoptosis in gastric cancer cells, with LINC00511 overexpression resulting in decreased apoptosis (Sun S. et al., 2020). Furthermore, ChIP and luciferase assays demonstrated that HIF-1α binds to the promoter region of lncRNA HYPAL, thereby promoting its expression in gastric cancer cells. HYPAL functions as a ceRNA for miR-431-5p, regulating the expression of CDK14. The HIF-1α/HYPAL/miR-431-5p/CDK14 axis activates the Wnt/β-catenin signaling pathway, thereby inducing proliferation in gastric cancer cells while inhibiting apoptosis (Piao et al., 2022). Moreover, lncRNA-NUTF2P3-001 has been reported to be upregulated in pancreatic cancer cells under hypoxic conditions and cobalt (II) chloride treatment, attributed to HIF-1α binding to the HRE upstream of the KRAS promoter. This study found that downregulation of NUTF2P3-001 using NUTF2P3-001-siRNA significantly reduced the viability of pancreatic cancer cells. Additionally, the proliferation of pancreatic cancer cells was markedly inhibited following treatment with lentivirus containing NUTF2P3-001-siRNA (LV-NUTF2P3-001-siRNA) (Li et al., 2016). These studies demonstrate that lncRNA-HIF interactions critically balance proliferative and apoptotic signals across digestive tumors, with dysregulation contributing to uncontrolled growth.

## Interaction between lncRNA and HIF regulates metastasis and invasion of digestive system tumors

Cancer metastasis refers to the dissemination of tumor cells from their primary site to distant regions of the body (Li et al., 2012). The processes of metastasis and invasion are significantly influenced by tumor-matrix interactions, including extracellular matrix (ECM) remodeling and EMT (Kai et al., 2016). Recent investigations into metastasis in digestive tumors have underscored the critical role of the interplay between lncRNAs and HIF-1α. Wang et al. demonstrated that the lncRNA DACT3-AS1 is transcriptionally activated by HIF-1α in HCC cells under hypoxic conditions. Furthermore, HDAC2 was identified as a co-binding protein of DACT3-AS1 and FOXA3, with DACT3-AS1 enhancing the metastatic function of HIF-1α in HCC cells by increasing the binding affinity between FOXA3 and HDAC2, which subsequently reduced FOXA3 expression. They observed that the migration, invasion, and EMT processes of HCC cells were impeded by the downregulation of DACT3-AS1, as demonstrated through wound healing and Transwell assays, while a decrease in FOXA3 yielded the opposite effect. In summary, DACT3-AS1 downregulates FOXA3 expression, thereby facilitating HCC cell migration and invasion, as well as EMT (Wang L. et al., 2022). In a separate assay involving hepatocellular carcinoma, the potential for cell migration and invasion was evaluated using Transwell assays, revealing that the knockdown of lncRNA MIAT or the absence of HIF-1α inhibited the proliferation, migration, and invasion of hypoxia-treated hepatoma cells. Additionally, the study indicated that X-ray analysis of HCC cells was insufficiently robust to detect the presence of HIF-1α (Liu et al., 2020). Furthermore, studies have established that XIST functions as an oncogene in colorectal cancer, with elevated levels of XIST positively correlating with tumor progression. XIST acts as a ceRNA for miR-93-5p, promoting colorectal cancer metastasis partially through HIF-1A/AXL signaling (Yang et al., 2020). PCGEM1 is a hypoxia-responsive lncRNA that is overexpressed in GC cells and tissues, and its expression is induced by hypoxia in GC cells. Studies have demonstrated that the knockdown of PCGEM1 significantly inhibits the invasion and metastasis of GC cells. SNAI1, a key transcription factor involved in EMT, is regulated by PCGEM1. Notably, the overexpression of SNAI1 can rescue the inhibitory effects caused by PCGEM1 knockdown during the invasion and metastasis of GC cells. Additionally, PCGEM1 and SNAI1 collectively influence the expression of EMT biomarkers (Zhang et al., 2019). Furthermore, lncRNA CF129 has been shown to enhance the interaction between p53 and the E3 ligase MKRN1, leading to the ubiquitination and degradation of p53 in pancreatic cancer. p53 promotes the transcription of FOXC2, which in turn regulates the transcription of HIF-1α. Consequently, CF129 indirectly inhibits the expression of HIF-1α. Therefore, CF129 can inhibit HIF-1α expression by inducing p53 instability and suppressing FOXC2 expression, thereby reducing the metastasis and invasion of pancreatic cells (Liu et al., 2019). Evidence converges that lncRNA-HIF axes serve as master regulators of EMT and ECM remodeling, providing mechanistic insights into metastatic dissemination in hypoxic microenvironments.

## Interaction between lncRNA and HIF regulates angiogenesis and immune escape in digestive system tumors

Angiogenesis is a multistep process of blood vessel neovascularization that supplies cells with oxygen and nutrients while facilitating the expulsion of metabolic wastes (Giordano et al., 2014). In the context of tumors, the heightened nutritional demands of tumor cells disrupt the equilibrium between pro-angiogenic and anti-angiogenic factors, thereby sustaining primary tumors and promoting metastasis to secondary sites (Carmeliet, 2005). Hypoxia is a well-established trigger for neovascularization. Recent studies have extensively investigated the mechanisms underlying angiogenesis in tumor cells, revealing the involvement of lncRNAs and HIF-1α in various stages of the angiogenic response. VEGF is recognized as a critical angiogenic factor (Ma et al., 2019). One study demonstrated that VEGF is a downstream target of the lncRNA PAARH in HCC, with VEGF expression showing a significant positive correlation with PAARH levels in HCC tissues, thereby promoting angiogenesis through VEGF upregulation (Wei et al., 2022). In another study, lncRNA UBE2CP3 in HCC cells was found to enhance VEGFA secretion into the supernatant by activating the ERK/HIF-1α signaling pathway, which in turn promotes endothelial cell (EC) proliferation, migration, and angiogenesis (Lin et al., 2018). Additionally, lncRNA SZT2-AS1 was identified as a novel lncRNA in HCC, which recruits HIF-1α and HIF-1β to form HIF-1 heterodimers. HIF-1 is essential for the transcriptional occupancy of HRE and HIF target genes, with SZT2-AS1 promoting HCC angiogenesis both *in vitro* and *in vivo* (Liu et al., 2024a). Tumor immune escape refers to the phenomenon whereby tumor cells proliferate and metastasize through various mechanisms to evade detection and attack by the immune system (You et al., 2021). In HCC, the expression levels of programmed cell death ligand 1 (PD-L1) and long non-coding RNA MIR155HG were significantly higher in patients exposed to hypoxic conditions compared to those in non-hypoxic conditions. HIF-1α binds to the promoter region of MIR155HG, thereby enhancing its transcriptional activity under hypoxic conditions. This hypoxic environment acts as a stressor that promotes the nuclear export of ILF3, which subsequently increases the binding affinity of ILF3 to MIR155HG. This interaction enhances the stability of HIF-1α mRNA. Consequently, hypoxia-induced interaction between MIR155HG and ILF3 leads to increased stability of HIF-1α mRNA, resulting in elevated PD-L1 expression in HCC and promoting immune escape (Qiu et al., 2024). Furthermore, long non-coding RNA NNT-AS1 is highly expressed in PC, and HIF-1α transcription activates the expression of NNT-AS1, which in turn enhances the stability and expression of integrin beta 1 (ITGB1) in a METTL3-HuR-dependent manner. Notably, overexpression of ITGB1 reversed the inhibitory effect of NNT-AS1 knockdown on hypoxia-induced immune escape in PC cells (Lu et al., 2023). Crucially, lncRNA-HIF circuits coordinate angiogenic switch and immunosuppression through VEGF/PD-L1 pathways, revealing dual targets for microenvironment modulation.

## Interaction between lncRNA and HIF regulates metabolism in digestive tumors

During hypoxia, the primary cellular metabolic strategy can rapidly shift from predominantly mitochondrial respiration to glycolysis in order to maintain ATP levels. This metabolic shift can be regulated by the expression of HIF-dependent and HIF-independent glycolytic enzymes (Al Tameemi et al., 2019; Kierans and Taylor, 2021). Several lncRNAs have been identified as regulators of this reprogramming, promoting cancer cell growth and invasion under hypoxic stress. For instance, lncRNA RAET1K is associated with HIF1A and miR-100-5p. Silencing of lncRNA RAET1K significantly inhibited the hypoxia-induced increases in lactate concentration and glucose uptake in HCC cells, whereas miR-100-5p mitigated the effects of lncRNA RAET1K silencing on hypoxia-induced glycolysis in HCC cells (Zhou et al., 2020). Additionally, He et al. reported that lncRNA NPSR1-AS1 is expressed in HCC cells, and its overexpression increases the levels of phosphorylated ERK1/2 (p-ERK1/2) and pyruvate kinase M2 (PKM2) in these cells. Knockdown of NPSR1-AS1 eliminated the hypoxia-induced activation of the MAPK/ERK pathway in HCC cells. Furthermore, depletion of NPSR1-AS1 partially reversed the effects of NPSR1-AS1 silencing on glycolysis and proliferation in hypoxia-induced HCC cells *in vitro*. These findings suggest that hypoxia-induced NPSR1-AS1 may promote proliferation and glycolysis in HCC cells by regulating the MAPK/ERK pathway (He et al., 2020). Tang et al. demonstrated that silencing the lncRNA CRNDE enhanced the expression of miR-142, decreased the levels of enhancer of zeste homolog 2 (EZH2) and HIF-1α. Furthermore, overexpression of HIF-1α mitigated the reduction in glucose depletion and lactic acid production induced by CRNDE silencing. Their investigation revealed that CRNDE knockdown inhibited glycolysis via the EZH2/miR-142/HIF-1α pathway (Tang et al., 2022). Additionally, lncRNA HOTAIR expression was found to be elevated in HCC tissues and cells under hypoxic conditions. HOTAIR was validated as a decoy for miR-130a-3p, with HIF1A identified as a target of miR-130a-3p. HOTAIR knockdown in hypoxia-treated HCC cells inhibited glycolysis through the regulation of miR-130a-3p and HIF1A (Hu et al., 2020). Similarly, lncRNA PVT1 was overexpressed in human pancreatic ductal adenocarcinomas (PDAC) and was associated with reduced patient survival. Knockdown of lncRNA PVT1 resulted in decreased glucose uptake, lactate secretion, intracellular ATP levels, and HIF-1A expression in pancreatic cancer cells. Results from luciferase reporter gene assays demonstrated a direct interaction between PVT1 and miR-519d-3p, indicating that miR-519d-3p may directly interact with and negatively regulate the expression of the 3′UTR mRNA of HIF-1A. Thus, it was concluded that upregulation of PVT1 promotes the progression of pancreatic ductal adenocarcinoma and glycolysis by regulating miR-519d-3p and HIF-1A (Sun J. et al., 2020). The data collectively indicate that lncRNA-HIF networks reprogram glycolytic flux and mitochondrial function to sustain bioenergetic demands of hypoxic tumor cells.

## Interaction between lncRNA and HIF in chemoresistance and targeted therapy of digestive system tumors

**FIGURE 2:**
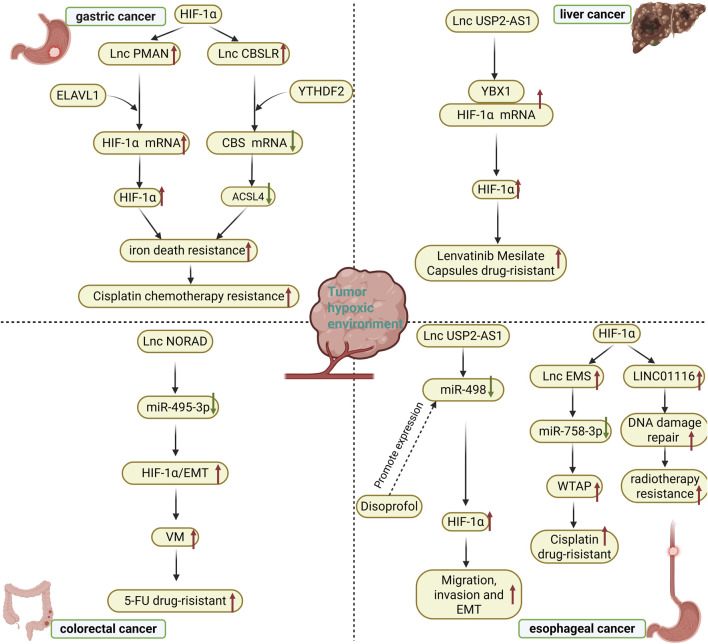
Chemoresistance and targeted therapy for digestive system tumors in hypoxic environments. ELAVL1: embryonic lethal abnormal vision like 1; YTHDF2: YTH N6-Methyladenosine RNA Binding Protein F2; ACSL4: Acyl-CoA Synthetase Long Chain Family Member 4; YBX1: The Y-box binding protein 1; VM: vasculogenic mimicry; 5-FU: 5-Fluorouracil; WTAP: Wilms’ tumor 1-associating protein; EMT: Epithelial-mesenchymal transition.

In gastrointestinal tumors, the interaction between long non-coding RNA (lncRNA) and HIF signaling has significant clinical implications, particularly as diagnostic biomarkers, prognostic indicators, and therapeutic targets, offering new perspectives for improving patient management. Therefore, we summarized all long non-coding RNAs mentioned in the full text that can be used as prognostic and diagnostic markers for these tumors (Table 5). For example, in gastric cancer, the transcription of lncRNA GAPLINC is directly regulated by HIF-1α activation, a mechanism associated with cancer progression, suggesting that GAPLINC has potential as a liquid biopsy tool to assist in guiding clinical treatment decisions (Liu et al., 2016). Additionally, multiple studies have found that lncRNA H19 is overexpressed in gastrointestinal tumors, participating in tumor development and chemotherapy resistance by regulating abnormal gene expression. Its expression levels are correlated with pathological features, making it a potential marker for predicting poor prognosis (Wang J. et al., 2021; Lin et al., 2016). Similarly, lncRNA MALAT1 is overexpressed in various gastrointestinal cancers and has been shown to be associated with poor prognosis, supporting its role as a reliable prognostic biomarker (Song et al., 2016).Furthermore, lncRNA LncHIFCAR (i.e., MIR31HG) is induced under hypoxic conditions and acts as a co-activator of HIF-1α to regulate key transcriptional networks, promoting tumor progression and metastasis. This highlights the clinical application potential of targeting the lncRNA-HIF axis, including reversing chemotherapy resistance (Shih et al., 2017; Miao et al., 2021). Studies have also shown that the expression of specific lncRNAs, such as lncRNA-ATB, is associated with patient prognosis in digestive system cancers, further reinforcing the role of lncRNAs in clinical classification and precision therapy (Duan et al., 2020). These findings collectively emphasize that targeting the lncRNA-HIF interaction axis (e.g., by inhibiting or silencing relevant lncRNAs) holds promise for overcoming chemotherapy resistance, enhancing drug sensitivity, and developing liquid biopsy-based diagnostic strategies, thereby improving the efficacy of personalized management of gastrointestinal tumors (Figure 2).

**TABLE 5:** Prognostic/diagnostic HIF-associated lncRNA biomarkers in digestive system tumors.

Tumor type	lncRNA	HIF regulation	Expression	Sample source	Clinical significance
Hepatocellular Carcinoma (HCC)	CPS1-IT1	Inhibits HIF-1α	Downregulated	Tissue	High expression correlates with prolonged OS; suppresses metastasis/EMT
	TRERNA1	Synergizes with HIF-1α	Upregulated	Tissue/Serum	Combined with HIF-1α predicts poor prognosis (Sensitivity: 82.4%, Specificity: 76.9%)
	PAARH	Activates HIF-1α	Upregulated	Tissue	Promotes angiogenesis; high expression shortens DFS
	MIR155HG	Stabilizes HIF-1α	Upregulated	Tissue	High expression increases PD-L1 positivity; predicts immunotherapy resistance
Colorectal Cancer (CRC)	NORAD	Activates HIF-1α	Upregulated	Tissue/Plasma	Mediates 5-FU resistance; high expression induces vasculogenic mimicry (predicts recurrence)
	CPS1-IT1	Inhibits HIF-1α	Downregulated	Tissue	Low expression enhances autophagy; associates with metastasis/advanced TNM staging
	CRNDE	Activates HIF-1α	Upregulated	Tissue	High expression upregulates glycolysis; predicts chemotherapy resistance
Gastric Cancer (GC)	GAPLINC	HIF-1α-induced	Upregulated	Tissue	High expression enriches CD44^+^ stem cells; predicts cisplatin resistance (Resistance index: ↑3.2-fold)
	BC005927	HIF-1α-induced	Upregulated	Tissue	Co-expressed with EPHB4; predicts lymph node metastasis (AUC = 0.84)
	NUTM2A-AS1	Modulates HIF-1α	Upregulated	Tissue	High expression increases PD-L1 positivity; predicts response to immune checkpoint inhibitors
Pancreatic Cancer (PC)	ENST00000480739	Inhibits HIF-1α	Downregulated	Tissue	Low expression correlates with advanced TNM stage/distant metastasis
	BANCR	Stabilizes HIF-1α	Upregulated	Tissue/Lymph fluid	Promotes lymphangiogenesis; high expression increases lymphatic metastasis (OR = 4.32)
	LINC00460	HIF-1α-induced	Upregulated	Tissue	Activates UBE2V1/p53 pathway; predicts early recurrence

Multidrug resistance and radioresistance continue to pose significant challenges to effective cancer treatment. Hypoxic cells generally exhibit greater chemo- and radio-resistance compared to normoxic cells (Yasuda, 2008), with the activation of HIF-1α-related pathways contributing to increased resistance in tumor cells (Comerford et al., 2002; Comerford et al., 2004; Moeller and Dewhirst, 2006). For instance, HIF-1α regulates the transcription of numerous genes associated with chemoresistance, such as ABC transporter protein genes, as well as those linked to radioresistance, including p53 and p21 (Yasuda, 2008). Chemo- and radio-resistant tumor cells are often reliant on various mutated genes that present potential therapeutic targets. Notably, the expression of lncRNA USP2-AS1 is significantly upregulated in hypoxic microenvironments, activating downstream hypoxic response pathways by enhancing the interaction between the RNA-binding protein YBX1 and HIF1α mRNA, thereby stabilizing HIF1α mRNA and increasing its protein levels. This USP2-AS1/HIF1α positive feedback loop facilitates migration, invasion, and the development of drug resistance in hepatocellular carcinoma cells. Importantly, the knockdown of USP2-AS1 markedly improved the therapeutic efficacy of the tyrosine kinase inhibitor lenvatinib and inhibited tumor growth in an *in vivo* model, indicating that targeting USP2-AS1 may reverse therapeutic resistance in hepatocellular carcinoma cells by disrupting HIF1α signaling (Chen et al., 2022). Zhang et al. demonstrated that CRC cells exposed to hypoxia exhibited an enhanced ability to form angiogenic mimics (VMs). The expression levels of lncRNA NORAD and HIF-1α were elevated in CRC tissues. NORAD functions as a miR-495-3p sponge, regulating HIF-1α-EMT signaling, while its increased expression influences hypoxia-induced HIF-1α signaling, thereby modulating VM formation and 5-FU drug resistance. The knockdown of NORAD reduced hypoxia-induced VM formation and VE-calmodulin expression. Furthermore, the downregulation of NORAD sensitized CRC cells to 5-FU by exacerbating the 5-FU-induced decrease in cell viability and enhancing apoptosis (Zhang et al., 2022). In response to hypoxia induction, lncRNA-CBSLR interacts with the YTH structural domain family protein 2 (YTHDF2) to form the CBSLR/YTHDF2/CBS signaling axis, which reduces the stability of CBS mRNA by enhancing the binding of YTHDF2 to the m6A-modified coding sequence (CDS) of CBS mRNA. Additionally, in the presence of reduced CBS levels, ACSL4 protein methylation decreases, leading to protein polyubiquitination and subsequent degradation of ACSL4. This process reduces the levels of phosphatidylethanolamine (PE) that promote iron death, thereby protecting GC cells from iron death and contributing to chemoresistance (Yang et al., 2022). Another experiment indicated that iron death is associated with chemoresistance in gastric cancer, where HIF-1α promotes its accumulation by regulating the expression of lncRNA-PMAN, thus forming the HIF-1α/PMAN/ELAVL1 regulatory axis. In this axis, ELAVL1, an RNA-binding protein, mediates the enhancement of PMAN’s effect on the stability of HIF-1α mRNA. This positive feedback mechanism enhances the resistance of gastric cancer cells to iron death in a hypoxic microenvironment, ultimately leading to chemoresistance (Lin et al., 2022). Furthermore, another study on gastric cancer revealed that HIF-1α directly activates the transcription of lncRNA GAPLINC, which promotes tumor invasion and drug resistance in gastric cancer by regulating stem cell-related genes such as CD44, suggesting that targeting GAPLINC may improve chemosensitivity (Liu et al., 2016). HIF signaling in hypoxic environments upregulates the expression of long-chain non-coding RNA lncRNA-EMS, which functions as a ceRNA by adsorbing miR-758-3p. This interaction disrupts the inhibitory effect of miR-758-3p on WTAP (Wilms’ tumor 1-associated protein), resulting in increased WTAP expression. Abnormally high levels of WTAP enhance cisplatin resistance in EC cells, ultimately leading to a hypoxia-HIF-lncRNA-EMS-WTAP-driven chemoresistance phenotype (Zhu et al., 2021). In another study on esophageal cancer, HIF-1α promoted the expression of LINC01116 by directly binding to its promoter region. This lncRNA mediated radiotherapy resistance by regulating the DNA damage repair pathway, while inhibition of HIF-1α or LINC01116 increased radiotherapy sensitivity (Zhang et al., 2024). Under hypoxic conditions, lncRNA TMPO-AS1 inhibited miR-498 expression by directly binding to it, thereby enhancing the activity of the HIF signaling pathway. This effect further promotes the migration, invasion, and epithelial-mesenchymal transition of esophageal cancer cells, ultimately leading to chemoresistance. Additionally, propofol can block the inhibitory effect on miR-498 by suppressing TMPO-AS1 expression, which in turn inhibits the HIF-induced malignant phenotype of the cells and enhances sensitivity to chemotherapy (Gao et al., 2020). This evidence positions lncRNA-HIF interactions as central mediators of treatment failure, while offering actionable targets for chemosensitization strategies in digestive oncology.

## Conclusions and future perspectives

Decades of intensive research on hypoxia biology and hypoxia-inducible factors have significantly enhanced our understanding of oxygen homeostasis. Recent years have witnessed a dramatic increase in our knowledge of the non-coding transcriptome, with HIF identified as a major regulator of both coding and non-coding transcriptomes (Choudhry et al., 2014; Ortiz-Barahona et al., 2010). Conversely, several lncRNAs have been shown to modulate HIF activity and stability, thereby adding an additional layer of complexity to the role of lncRNAs in gene expression regulation during hypoxia. In this review, we summarize the available evidence for bidirectional interactions between HIF-1α and lncRNAs in digestive system tumors. We address how lncRNAs interact with HIF-1α, influencing tumor progression through the regulation of various biological functions. This highlights the complex relationship among lncRNAs, hypoxia, and their target genes in the context of chemotherapy resistance. In the treatment of gastrointestinal tumors, lncRNAs demonstrate unique advantages, such as their tissue-specific expression, which can serve as diagnostic markers and potential intervention targets. For example, LINC00665 is abnormally expressed in tumors such as gastric cancer and colorectal cancer and influences prognosis (Yuan et al., 2024). Additionally, they can dynamically regulate tumor progression by affecting multiple signaling pathways, such as the PI3K/AKT pathway, in response to cellular stress environments (Chen et al., 2018), and can be utilized as therapeutic targets through RNA-based therapies like antisense oligonucleotides (ASOs) (Liu et al., 2024b). However, the application of lncRNAs faces significant limitations: the lack of delivery systems is a major obstacle, the insufficient sensitivity of liquid biopsy and other bodily fluid tests limits their practicality as biomarkers, and functional redundancy must be overcome through multi-target combination strategies to ensure effective regulation of complex tumor mechanisms (Li et al., 2024). A comprehensive understanding of these interactions is crucial for developing new therapeutic approaches to combat drug resistance in the treatment of digestive system tumors. By targeting these lncRNAs, it may be possible to overcome drug resistance in cancer patients, indicating their potential as effective therapeutic targets and chemotherapeutic enhancers. However, this field is still in its early stages, and some of the latest research requires further refinement. For example, the micropeptide AC115619-22aa encoded by the long non-coding RNA AC115619 is downregulated in liver cancer tissues. This micropeptide reduces m6A levels by disrupting the formation of the m6A methylation complex, thereby inhibiting the proliferation and progression of liver cancer cells. It has the potential to serve as a prognostic marker and therapeutic target for liver cancer (Zhang Q. et al., 2023). In contrast, the micropeptide SMIM30 encoded by LINC00998 promotes the proliferation, migration, and metastasis of liver cancer cells by interacting with the ribosomal protein RPS6, and its high expression is associated with poor prognosis in liver cancer patients (Pang et al., 2020). These two types of lncRNA-derived micropeptides exhibit distinct roles in regulating hepatocellular carcinoma progression: the micropeptide encoded by AC115619 exhibits anticancer activity, while SMIM30 exerts pro-cancer functions. Future studies may further explore the interaction mechanisms between such micropeptides and the hypoxia-inducible factor HIF-1α pathway in gastrointestinal tumors.Numerous challenges remain, including the validation of newly discovered lncRNAs and their functions under hypoxic conditions, the development of novel therapeutic and prophylactic drugs targeting lncRNAs, and the exploration of the translational potential of these hypoxia-associated lncRNAs for cancer treatment. While most lncRNA-targeted therapies are still in early development stages, future technological advancements and deeper insights into lncRNA pathways in cancer biology will offer new opportunities. Our review also aims to enhance understanding of the regulatory role of lncRNAs in the tumor microenvironment and to facilitate the development of novel anticancer drugs.
