# Correction: Crosstalk between hypoxia-inducible factor (HIF) and lncRNAs in digestive tumors: from molecular mechanisms to clinical translation

**DOI:** 10.3389/fcell.2025.1684892

**Published:** 2025-09-10

**Authors:** Lifeng Gan, Peiyue Luo, Junrong Zou, Wei Li, Qi Chen, Le Cheng, Fangtao Zhang, Haidong Zhong, Yiran Lu, Liying Zheng, Biao Qian

**Affiliations:** ^1^ The First Clinical College, Gannan Medical University, Ganzhou, Jiangxi, China; ^2^ Department of Urology, The First Affiliated hospital of Gannan Medical University, Ganzhou, Jiangxi, China; ^3^ Department of Urology and Andrology, The Key Laboratory of the First Clinical Medical College of Gannan Medical University, Ganzhou, Jiangxi, China; ^4^ Department of Graduate, The First Affiliated Hospital of Gannan Medical University, Ganzhou, Jiangxi, China

**Keywords:** hypoxia, HIF, lncRNA, digestive system tumors, liver cancer, colorectal cancer, gastric cancer, pancreatic cancer

There was a mistake in Figure 1 as published. The oxygen concentration labels for “Normoxia” and “Hypoxia” were inadvertently swapped. The original figure incorrectly labeled: Normoxia as “0.1%–1%” and Hypoxia as “21%”. The corrected Figure 1 appears below.

**FIGURE 1:**
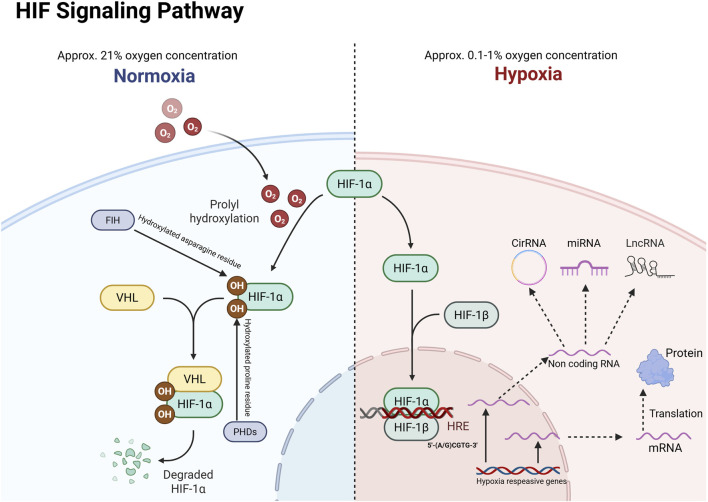
HIF-1α responds to gene transcription under hypoxic activation. VHL: Von Hippel-Lindau; HRE: hypoxia response element; PHD: Prolyl hydroxylase; FIH: Factor-inhibiting HIF.

The original article has been updated.

